# Flavor Composition and Microbial Community Structure of Mianning Ham

**DOI:** 10.3389/fmicb.2020.623775

**Published:** 2021-01-26

**Authors:** Lin Chen, Zhengli Wang, Lili Ji, Jiamin Zhang, Zhiping Zhao, Rui Zhang, Ting Bai, Bo Hou, Wei Wang

**Affiliations:** Key Lab of Meat Processing of Sichuan Province, Chengdu University, Chengdu, China

**Keywords:** Mianning ham, volatile flavor compound, high-throughput sequencing, fungal community diversity, colony screening, quality

## Abstract

Mianning ham, a traditional Chinese dry-cured ham, is protected by national geographical indications. To understand the surface and internal flavor composition and microbial community structure of Mianning ham, solid phase microextraction-gas chromatography (SPME-GC-MS) technology and Illumina high-throughput sequencing were utilized. The results showed that a total of 60 flavor substances were identified in the hams. Forty-nine kinds of flavorings were identified on the surface, including 14 aldehydes, 6 ketones, 10 alcohols, 5 esters, 7 hydrocarbons, 5 acids, and 2 other compounds. Thirty-six kinds of internal flavorings were identified, including 13 aldehydes, 4 ketones, 6 alcohols, 3 esters, 5 hydrocarbons, 4 acids and 1 other type. Decanal (34.91 μg/g) was the most prevalent compound on the surface, followed by n-hexanol (24.99 μg/g), n-hexanal (20.20 μg/g), and n-octyl (16.14 μg/g). n-Hexanal (20.74 μg/g) was the most common compound internally, followed by non-aldehyde (5.70 μg/g), 1-octene-3-alcohol (3.54 μg/g), and inverse-2-octenal (2.77 μg/g). *Penicillium lanosum, Penicillium nalgiovense, Debaryomyces hansenii, Staphylococcus equorum*, and *Erwinia tasmaniensis* were isolated from the surfaces of the hams by the traditional culture method. By Illumina high-throughput sequencing, three fungal phyla were identified. Ascomycota was the dominant phylum followed by Basidiomycota. At the genus level, 11 fungi were identified, of which *Aspergillus* was the dominant fungus, followed by *Penicillium* and *Wallemia*. These findings provide fundamental knowledge regarding the microorganisms and flavor compounds in Mianning ham, which will help industrial processors develop effective strategies for standardizing quality parameters.

## Introduction

Mianning ham is a famous fermented meat product in Mianning, Sichuan province in southwest China, and is protected by national geographical indications. Mianning hams are made by traditional local techniques and are salty, delicious, rich in aroma, and bright in color. Mianning ham is characterized by plump muscles and small legs, generally weighing 4 ∼ 6 kg (Yang, 1979). The production technology of Mianning ham includes material selection, repair, curing, washing, and drying, fermentation, and storage. In the production process, plump, fresh, and healthy hind legs should be selected. After four salt applications, the total amount of salt is 9∼10% of the leg weight. After salting, the surface oil and excess salt are washed with clean water, the ham is hung in a ventilated area indoors, and natural fermentation is allowed to occur (Yu, 2004).

Flavor is an important index to evaluate the quality of ham. The flavor of ham comes from the oxidative decomposition of endogenous enzymes during natural fermentation and extracellular enzymes and metabolites secreted by microorganisms (Harkouss et al., 2015; Mu et al., 2019). The microorganisms in ham mainly come from the meat itself, auxiliary materials that are added, the processing environment and manual contact (Comi et al., 2004) and include lactobacillus, staphylococcus, micrococcus, mold, and yeast (Wang, 2008). Microorganisms play an important role in the formation of ham flavor. Microorganisms can degrade the protein of ham muscle and promote the formation of its quality characteristics. At present, there is no report on the flavor composition and microorganisms of Mianning ham. Therefore, the study of the surface and internal flavor composition and microbial community structure of these hams is of great significance for theoretical research and development of the Mianning ham industry.

## Materials and Methods

### Sample Preparation and Sampling

Ten Mianning hams were selected from a batch of hams produced at a processing plant in Mianning (China) and ripened for 24 months. A 0.2-cm-thick piece of Mianning ham surface meat was cut with a sterile scalpel and taken as the surface (BM) sample. Then, a 1 ∼ 1.5-cm-thick piece of ham surface was removed with a scalpel, and sterile scissors or scalpels were used to collect a meat sample from a depth of 3 ∼ 4 cm as an internal (NB) sample (Zhen, 2004). Then, the samples were stored at −20°C for subsequent analysis.

### Physical and Chemical Index Measurements

The pH and a_w_ were measured in triplicate according to the methods described by Wang et al. (2018). The water, nitrite, malondialdehyde, protein, and fat contents were measured in triplicate according to the methods described by Li et al. (2020). The *L*^∗^ (brightness), *a*^∗^ (redness), and *b*^∗^ (yellowness) values were measured in triplicate according to the methods described by Pan (2016).

### Flavor Compound Analysis

The surface and internal samples of Mianning ham were cut into pieces, and 3.00 g was accurately weighed into a 15 mL headspace bottle. Then, 1 μL 2,4,6-trimethylpyridine was added to the headspace bottle as an internal standard, and the bottle was sealed (Bai et al., 2018; Zhang et al., 2020). The extraction method for the flavoring substances was headspace solid-phase microextraction (SPME). The pretreatment conditions of the sample were set by the CTC automatic sampler: the heating chamber temperature was 75°C, the heating time was 45 min, the sample extraction time was 20 min, and the analysis time was 5 min. The flavor substances were analyzed with gas chromatography-mass spectrometry (GC-MS).

Gas chromatography conditions: an HP-5 MS UI chromatographic column (30 m × 0.25 mm, 0.25 m) was used; the pressure was 32.0 kPa; the column flow velocity was 1.0 mL/min; the carrier gas was helium, and splitless injection was performed. The temperature of the injection port was 250°C.

Column temperature program: the starting temperature was 40°C for 15 min; the temperature was increased to 160°C at 3°C/min and maintained for 0 min; then, the temperature was increased to 230°C at 4°C/min and maintained for 5 min.

Mass spectrometry conditions: an electron ionization source (EI) was used; the electron energy was 70 eV; the ion source temperature was 230°C; the temperature of the four-stage rod was 150°C; the quality scanning range was 35 ∼ 500 m/z; and the detector voltage was 350 V.

Qualitative analysis: the resulting sample chromatograms were integrated, and the volatile compounds corresponding to the peaks on the matched chromatogram were retrieved from the NIST14. L library at a matching degree of 80%.

Quantitative analysis: the relative contents of each component were obtained by normalizing the peak areas of the total ion flow chromatograms.

### Microbial Separation Count

Under aseptic conditions, 12.5 g surface and internal samples were weighed and placed in a sterile homogenizer, 112.5 mL sterile normal saline was added, and the homogenizer was used to beat the samples for 4 min at 5 T/sec to make a 10^–1^ diluent. Then, bacterial solutions of 10^–2^, 10^–3^, and 10^–4^ were successively obtained according to the 10-fold dilution method. Then, 0.1 ml of each bacterial dilution was inoculated with the smear method into the following media: plate count agar medium (PCA) with incubation at 36°C for 48 h; MRS agar medium at 36°C for 72 h; mannitol fermentation medium at 36°C for 24 h; and bengal red culture medium at 28°C for 5 days.

### Isolation and Identification of Microorganisms

Plates with microbial community numbers between 30∼300 CFU were selected, and a single colony was selected to be smeared and purified on a sterilized plate for growth. The culture temperature and time were the same as before. Individual colonies were observed and preliminarily identified by microscopy after Gram staining. After the preliminary identification, the characteristic colonies were selected for further culture.

The genomic DNA of bacteria and fungi was extracted according to the instructions of the genomic DNA extraction kit. The bacterial 16S rRNA gene was amplified with universal primers: upstream primer 27F (5′-AGAGTTTGATCCTGGCTCAG-3′) and downstream primer 1492R (5′-GGTTACCTTGTTACGACTT-3′). The general primers for fungal ITS gene amplification were as follows: upstream primer ITS1 (5′-TCCGTAGGTGAACCTGCGG-3′) and downstream primer ITS4 (5′-TCCTCCGCTTATTATTGA TATGC-3′). The PCR mixture (50 μL) was mixed for 30 s and contained the following: Taq PCR Master Mix 25 L, template DNA 2 μL, primers 2 μL each, and sterile ddH_2_O 19 μL. PCR conditions were as follows: predenaturation at 94°C for 4 min; followed by 30 cycles of denaturation at 94°C for 30 s, annealing at 45°C for 30 s, and elongation at 72°C for 90 s; and final elongation at 72°C for 10 min. The PCR amplification products were determined by 1% agarose gel electrophoresis and then pooled on the device for sequencing. BLAST was used to analyze the homology of genes through the NCBI database, and the sequences of different species with high homology with the target gene sequences were obtained. The bacterial colony characteristics and morphology were comprehensively considered to confirm the identities of the strains.

### Fungal Diversity Analysis

Total microbial DNA of the samples was extracted with the HiPure Soil DNA kit according to the manufacturer’s protocol. Then, the DNA concentration and purity were determined by a NanoDrop microspectrophotometer. The extraction quality of DNA was determined by 2% agarose gel electrophoresis. The purified DNA was used for amplification of fungal ITS genes. ITS 3-kyo2 (F) (5′-GATGAAGAACGYAGYRAA-3′) and ITS4 (R) (5′-TCCTCCGCTTATTATTGATATGC-3′) were used as primers for PCR amplification of the fungal ITS2 region.

The first round PCR amplification system (50 μL) included the following: 5 μL 10 × KOD buffer, 5 μL 2 mM dNTPs, 3 μL 25 mM MgSO_4_, 1.5 μL 10 μM forward primer, 1.5 μL 10 μM reverse primer, 1 μL KOD enzyme, 100 ng DNA template, and distilled water to final volume. The amplification procedure was as follows: predenaturation at 94°C for 2 min, 30 cycles of denaturation at 98°C for 10 s, annealing at 62∼66°C for 30 s, and extension at 68°C for 30 s, and final extension at 68°C for 5 min. PCR products were purified by AMPure XP Beads. After purification, the PCR products were quantified with Qubit 3.0. The second round PCR amplification system (50 μL) contained the following: 5 μL 10 × KOD buffer, 5 μL 2 mM dNTPs, 3 μL 25 mM MgSO4, 1 μL 10 μM connector primer, 1 μL 10 μM PCR universal primer, 1 μL KOD enzyme, and 100 ng DNA template. The reaction was brough to final volume with distilled water. The amplification procedure was as follows: predenaturation at 94°C for 2 min, 30 cycles of denaturation at 98°C for 10 s, annealing at 65°C for 30 s, and extension at 68°C for 30 s, and final extension at 68°C for 5 min.

AMPure XP beads were used to purify the second round of amplification products. ABI StepOnePlus Real-Time PCR System (Life Technologies, origin United States) was used for quantitative analysis. According to Novaseq 6000 PE 250 model pooling, PCR amplification products were pooled on the device used for sequencing (Hirokazu et al., 2012). The QIIME platform and RDP Classifier were used to compare the representative OTU sequences, and the Silva taxonomy database was used to annotate the species classification information. The composition of each sample community was calculated according to the comparative data. The species diversity of samples was determined by alpha diversity analysis (Wang et al., 2019). The Ace, Chao1, Shannon and Simpson indexes of each sample at the 97% similarity level were evaluated by using Mothur software.

### Statistical Analysis

Microsoft Excel 2019 was used for data statistics, and IBM SPSS 25.0 was used for *t*-tests of the variance. The results showed that *P* > 0.05 was not significant. 0.05 > *P* > 0.01 was significant and marked with ^∗^; *P* < 0.01 was extremely significant and marked with ^∗∗^.

## Results and Discussion

### Physical and Chemical Index Analyses

The physical and chemical indexes of the surface (BM) and internal (NB) of Mianning ham are shown in Table 1. Physical and chemical properties are closely related to the quality of ham. Studies have shown that when the pH value of ham is >6.0 ∼ 6.2, there is a high risk of microbial contamination (Ramona et al., 2018); when the pH value is <6.0, the ham has an ideal texture and color; and when the pH value is <5.6, the ham has a more acidic taste (Gou et al., 2008). The surface pH value of the Mianning ham was 5.76 ± 0.01, and the internal pH value was 5.77 ± 0.02, which was close to the pH value of Panxian ham (5.73 ± 0.02) (Mu et al., 2020). The pH value plays an important role in presenting the umami taste of ham and has an obvious effect on the formation of flavor (Ma, 2014). During the processing, due to the REDOX reaction of myoglobin, the *L*^∗^ (brightness) of the surface was significantly lower than internal, the *a*^∗^ (redness) of the surface was extremely significantly lower than internal, and there was no significant difference in *b*^∗^ (yellowness). Because the ham of Mianning is preserved by hanging naturally, the tissue of the ham is hard outside and soft inside. The surface moisture content was 22.6 ± 0.681 g/100 g, and the internal moisture content was 36.7 ± 0.889 g/100 g. Thus, the surface moisture content was significantly lower than the internal moisture content. A low moisture content is of great significance to prevent spoilage and improve the quality of ham. Fan et al. (2019) found that the moisture content of Jinhua ham was 39.1 ± 0.37 g/100 g, that of Xuanwei ham was 45.2 ± 0.79 g/100 g, that of Rugao ham was 41.1 ± 0.09 g/100 g, and that of Xuanwei ham was 43.1 ± 0.12 g/100 g. In contrast, the moisture content of the Mianning ham was relatively low. Low water activity is conducive to the preservation of ham; when a_W_ < 0.85, most of the bacteria stop growing, while mold and a small amount of salt-consuming bacteria can also grow; the surface a_W_ of Mianning ham was 0.735 ± 0.001, and the internal a_W_ was 0.772 ± 0.004. Zhang et al. (2009) found that the a_W_ of Xuanwei ham was 0.758, which was close to the a_W_ of Mianning ham in this study. The content of fat and protein on the surface was 39.0 ± 1.0 g/100 g and 32 ± 0.58 g/100 g, respectively, and the content of fat and protein internal was 19.6 ± 1.1 g/100 g and 24 ± 3.1 g/100 g, respectively. The content of fat and protein on the surface was significantly higher than internal. The content of malondialdehyde on the surface of Mianning ham was 5.4 ± 0.31 mg/kg, and the content of malondialdehyde inside the ham was 1.7 ± 0.10 mg/kg. Malondialdehyde is the final product of fat oxidation. The surface of the ham had sufficient contact with oxygen, which made it more easily oxidized. The nitrite contents on the surface and internal areas of Mianning ham were 6.3 ± 0.58 and 0.3 ± 0.00 mg/kg, respectively, which were all within the range of nitrite residue limits (<30 mg/kg) of meat products in China. The nitrite content on the surface was significantly higher than the internal nitrite content. The reason for this finding may be that the pickling materials were all applied on the surface of the ham; only part of the nitrite enters into the ham, and the nitrite that enters into the ham is further degraded under the action of lactic acid (Guo, 2017). Dang found that the nitrite content of Nuodeng ham was 0.2 mg/kg after 1 year, close to the internal nitrite content of Mianning ham (Dang, 2018). Fan et al. (2019) found that the nitrite content of Xuanwei ham was 5.97 ± 0.05 mg/kg, close to the surface value of the Mianning ham.

**TABLE 1:** Physical and chemical indexes of Mianning ham.

Physicochemical indexes	BM	NB
pH	5.76 ± 0.01	5.77 ± 0.02
**Color deviation**		
L*	37.21 ± 0.28	41.82 ± 0.29*
a*	2.76 ± 0.41	11.67 ± 0.71**
b*	7.82 ± 1.37	10.38 ± 1.01
Moisture content (g/100 g)	22.6 ± 0.681	36.7 ± 0.889**
a_w_	0.735 ± 0.001	0.772 ± 0.004**
Fat (g/100 g)	39.0 ± 1.0	19.6 ± 1.1**
Protein (g/100 g)	32 ± 0.58	24 ± 3.1**
Malondialdehyde (mg/kg)	5.4 ± 0.31	1.7 ± 0.10**
Nitrite (mg/kg)	6.3 ± 0.58	0.3 ± 0.00**

BM is the surface sample of Mianning ham, NB is the internal sample of Mianning ham (the same below). Variance *t*-test: *P* > 0.05 was not non-significant, 0.05 > *P* > 0.01 was significant and marked with *; *P* < 0.01 was extremely significant and marked with **.

### Flavor Compound Analysis

The flavor compound data from the Mianning hams are shown in Table 2. Using the SPME-GC-MS method, 49 species flavor compounds were detected on the surface, including 14 aldehydes, 6 ketones, 10 alcohols, 5 esters, 7 hydrocarbons, 5 acids, and 2 other compounds. Thirty-six compounds were detected internally: 13 aldehydes, 4 ketones, 6 alcohols, 3 esters, 5 hydrocarbons, 4 acids, and 1 other compound. There were more kinds of flavor compounds on the surface than internal areas, among which aldehydes and alcohols were the predominant flavor compounds in Mianning ham. Decanal (34.91 μg/g) was the most abundant at the surface, followed by 1-hexanol (24.99 μg/g), hexanal (20.20 μg/g), and octanol (16.14 μg/g). Hexanal (20.74 μg/g) was the most abundant internally, followed by nonanal (5.70 μg/g), 1-octen-3-ol (3.54 μg/g), and (E)-2-octenal (2.77 μg/g). The types of flavor compounds on the surface were more abundant than those in the internal area. The reasons for this difference may be associated with the degree of oxidation on the surface. The surface is in full contact with air, and the degree of oxidation is high (the malondialdehyde content is high). Therefore, a wide variety of flavor substances are formed. The amount of each flavor substance relative to the standard substances was calculated, according to the amount of standard substance added to the ham and compared with the chromatographic peak area of flavor compounds and standard substances. The results showed that the contents of flavor compounds differed among the surface and internal areas. The reason for this difference may be related to the different degrees of oxidation between the surface and internal. A total of 60 flavor compounds were detected in the Mianning hams, and there were 26 common flavor compounds on the surface and internal areas. Using the SPME-GC-MS method, Mu et al. (2019) detected 51 kinds of flavor compounds in Panxian ham, and Tan et al. (2019) detected 56 kinds of flavor compounds in Jinhua Jinzi ham, 53 kinds of flavor compounds in Xuanwei Puji ham, and 57 kinds of flavor compounds in Changshou Rugao ham.

**TABLE 2:** Types and contents of flavor compounds in Mianning ham.

Compound	RT	Name	CAS	Absolute content (μg/g)	
				BM	NB
**Aldehydes**					
1	4.684	Hexanal	66-25-1	20.20 ± 4.44	20.74 ± 1.61
2	10.128	Heptanal	111-71-7	6.73 ± 1.02	1.88 ± 0.30
3	15.741	Benzaldehyde	100-52-7	0.48 ± 0.12	0.64 ± 0.03
4	20.59	Octanal	124-13-0	16.14 ± 1.98	2.44 ± 0.22
5	23.441	Benzeneacetaldehyde	122-78-1	1.92 ± 0.06	1.29 ± 0.34
6	24.774	(E)-2-Octenal	2548-87-0	3.27 ± 0.14	2.77 ± 0.54
7	27.946	Nonanal	124-19-6	34.91 ± 3.23	5.70 ± 1.19
8	31.059	(E)-2-Nonenal	18829-56-6	5.01 ± 0.36	1.98 ± 0.34
9	33.588	Decanal	112-31-2	8.79 ± 1.31	0.26 ± 0.13
10	33.897	(E,E)-2,4-Nonadienal	5910-87-2	0.90 ± 0.34	0.89 ± 0.51
11	36.339	(E)-2-Decenal	3913-81-3	7.28 ± 0.03	2.02 ± 0.76
12	37.843	2,4-Dodecadienal	13162-47-5	0.62 ± 0.10	–
13	38.869	(E,E)-2,4-decadienal	25152-84-5	1.84 ± 0.90	1.76 ± 0.11
14	40.921	Dihydro-5-pentyl-2(3H)-furanone	104-61-0	4.05 ± 0.14	0.34 ± 0.01
15	41.037	2-Undecenal	2463-77-6	4.52 ± 0.21	–
16	58.214	Hexadecanal	629-80-1	–	0.75 ± 0.03
**Ketones**					
17	9.359	2-Heptanone	110-43-0	6.67 ± 0.25	–
18	9.376	5-Methyl-2-hexanone	110-12-3	–	1.20 ± 0.22
19	19.634	2-Octanone	111-13-7	2.06 ± 0.17	–
20	25.743	3,5-Octadien-2-one	38284-27-4	–	2.16 ± 0.51
21	26.652	8-Nonen-2-one	5009-32-5	–	0.27 ± 0.08
22	27.165	3,5-Octadien-2-one	38284-27-4	–	2.36 ± 0.05
23	27.196	2-Nonanone	821-55-6	4.58 ± 1.98	–
24	35.174	1,4-Cyclooctanedione	55794-45-1	0.51 ± 0.05	–
25	36.462	Cyclodecanone	1502-06-3	0.56 ± 0.06	–
26	41.486	3,6-Dimethyl-octan-2-one	118452-32-7	0.36 ± 0.03	–
**Alcohols**					
27	8.100	1-Hexanol	111-27-3	24.99 ± 0.57	2.23 ± 0.37
28	17.874	1-Heptanol	111-70-6	4.18 ± 1.91	0.63 ± 0.03
29	18.644	1-Octen-3-ol	3391-86-4	1.78 ± 0.16	3.54 ± 0.10
30	25.772	(Z)-2-Nonen-1-ol	41453-56-9	1.08 ± 0.20	–
31	28.208	Phenylethyl Alcohol	60-12-8	1.07 ± 0.76	–
32	31.968	(S)-(+)-5-Methyl-1-heptanol	57803-73-3	4.33 ± 0.96	–
33	35.634	Z-2-Dodecenol	69064-36-4	–	0.23 ± 0.04
34	36.462	2-Methyl-1-decanol	18675-24-6	–	0.26 ± 0.04
35	38.781	3,4-Di[1-butenyl]-tetrahydrofuran-2-ol	1000131-84-0	0.35 ± 0.08	–
36	40.035	4,4,6-Trimethyl-cyclohex-2-en-1-ol	1000144-64-7	3.22 ± 0.32	0.66 ± 0.20
37	42.646	2-Butyl-1-octanol	3913-02-8	1.59 ± 1.49	–
38	46.056	E-2-Hexadecacen-1-ol	1000131-10-1	0.61 ± 0.00	–
**Ester**					
39	24.950	n-Caproic acid vinyl ester	3050-69-9	−	1.83 ± 0.58
40	25.985	Formic acid, octyl ester	112-32-3	5.67 ± 0.45	0.90 ± 0.14
41	34.736	Nitric acid, nonyl ester	20633-13-0	0.49 ± 0.11	–
42	36.077	5-Butyldihydro-2(3H)-furanone	104-50-7	1.14 ± 0.10	–
43	38.193	Heptadecyl prop-1-en-2-yl ester carbonic acid	1000382-90-2	0.43 ± 0.09	–
**Hydrocarbons**					
44	3.815	2-(1,1-Dimethylethyl)-3-methyl-oxirane	53897-30-6	1.75 ± 0.09	1.41 ± 0.04
45	15.951	1-Chloro-octane	111-85-3	2.49 ± 0.55	–
46	22.450	3-Ethyl-2-methyl-1,3-hexadiene	61142-36-7	–	0.35 ± 0.06
47	35.739	(Z)-5-Tridecene	25524-42-9	0.49 ± 0.03	–
48	38.513	Decyl-oxirane	2855-19-8	0.36 ± 0.17	–
49	41.043	1-Octadecyne	629-89-0	–	2.23 ± 0.05
50	42.605	2-(1-Methylpropyl)-bicyclo[2.2.1]heptane	74663-93-7	–	0.71 ± 0.13
51	45.245	2-Methyltetracosane	1560-78-7	0.46 ± 0.12	–
52	46.784	Pentadecane	629-62-9	2.13 ± 0.13	0.22 ± 0.07
53	50.672	Hexadecane	544-76-3	0.84 ± 0.04	–
**Acids**					
54	22.176	Hexanoic acid	142-62-1	1.24 ± 0.42	1.76 ± 0.88
55	27.631	Heptanoic acid	111-14-8	0.74 ± 0.17	–
56	32.964	Octanoic acid	124-07-2	2.95 ± 1.26	1.28 ± 0.30
57	37.353	Nonanoic acid	112-05-0	1.30 ± 0.07	0.32 ± 0.03
58	41.830	n-Decanoic acid	334-48-5	4.14 ± 0.22	1.04 ± 0.26
**Other**					
59	7.429	Syn-3-methyl-butyl aldoxime	5780-40-5	0.37 ± 0.03	–
60	19.448	2-pentyl-Furan	3777-69-3	4.43 ± 0.58	0.74 ± 0.04
61	19.693	2,4,6-trimethyl-Pyridine	108-75-8	6.67 ± 0.00	6.67 ± 0.00

*The symbol “–” denotes the substance was not detected.*

A large number of studies have found that the complex flavor system of meat is composed of components with flavor and aroma activity (Donald, 1998). Volatile flavor substances determine the flavor characteristics of ham and can be divided into two categories: the first class is simple compounds such as hydrocarbons, alcohols, aldehydes, ketones, acids, esters, etc.; the other is heterocyclic compounds containing oxygen, sulfur, and nitrogen atoms, such as furan and its derivatives, thiophene and its derivatives, etc. (Li et al., 2016). The formation pathways of ham flavor compounds include the decomposition of sulfur-containing amino acids, fatty acids, thiamine, the Maillard reaction between sugars and amino acids, microbial action, etc. (Guo et al., 2019; Zhang et al., 2019).

### Microbial Separation Count

After pure culture of surface and internal samples, it was found through plate counting results (Table 3) that the number of microorganisms on the surface was above 10^6^ CFU/g. The number of molds and yeasts was the largest and played an important role in the formation of ham flavor and quality. This is consistent with the research results of Li et al. (2003), in which the number of *Staphylococcus*, *Micrococcus*, and molds on the surface of Xuanwei ham was above 10^6^ CFU/g. In this experiment, the internal samples were examined twice according to the microbial culture standard in China, but no microorganisms were cultured. It may be that the high salt content (9∼10%) and low a_W_ in the ham inhibited the growth of microorganisms.

**TABLE 3:** Microbes in Mianning ham.

	Colony number (CFU/g)	Lactic acid bacteria (CFU/g)	Micrococcus and Staphylococcus (CFU/g)	Mold and yeast (CFU/g)
BM	2.4 × 10^6^	1.5 × 10^6^	2.1 × 10^6^	2.8 × 10^6^
NB	–	–	–	–

*The symbol “–” denotes the microbes was not detected.*

### Isolation and Identification of Microorganisms

Plate count agar, MRS, mannitol, and Bengal red plates were used to isolate and culture the characteristic bacteria from the ham surfaces, and a total of five strains were isolated and numbered MN1, MN2, MN3, MN4, and MN5. After observation of the presence of a single colony, Gram staining was used, and the colonies were then examined under the microscope. The bacterial colony characteristics and morphology are shown in Table 4. The isolated bacteria were all round colonies with neat middle raised edges. MN1 and MN2 had broom-like branches and were preliminarily determined to be molds. MN4 was a G^+^ bacteria, with a white color and a grape-like pattern and was preliminarily determined to be Staphylococcus.

**TABLE 4:** Colony characteristics and morphology of isolated strains from Mianning ham.

Name	Colony characteristics	Mycelial morphology
MN1	White, round, middle raised edge is neat	Broom-like branches, three-whorled, double-whorled or irregular, conidia spherical
MN2	Grayish white, round, middle raised edge is neat	Broom-like branches, three-whorled, double-whorled or irregular, conidia spherical
MN3	Reddish, round, middle raised edge is neat, matte	The bacteria are large, oval in shape
MN4	Milky white, round, smooth surface, middle raised edge is neat	G^+^, globular, singly, in pairs or in a grape-like pattern
MN5	White, smooth surface, round, middle raised edge is neat	G^–^, short rod-shaped, singly, in pairs or in irregular clumps

The isolated bacteria were subjected to ITS and 16S rRNA sequencing, and the identification results (Table 5) showed that MN1 was *Penicillium lanosum*, MN2 was *Penicillium nalgiovense*, MN3 was *Debaryomyces hansenii*, MN4 was *Staphylococcus equorum*, and MN5 was *Erwinia tasmaniensis*. *P. nalgiovense* is a rare species found in Shennongjia of Hubei province, Deyang of Sichuan province, Dujiangyan of Sichuan province, and Guiyang of Guizhou province. It has been reported from moldy pork, soil, pot lids and paper boxes. He et al. (2008) found that the molds in Jinhua ham mainly included *Penicillium italium*, *Penicillium simum*, *Penicillium citrate*, *Aspergillus sagrada*, and *Aspergillus flavus.* Zou et al. (2020) found that fungi in Jinhua ham mainly included *Aspergillus pseudoglaucus*, *Aspergillus penicillioides*, *Phialosimplex caninu*s, *Yamadazyma triangularis*, *Candida glucosophila*, *Wallemia sebi*, etc. Dang (2018) found that the fungi on the surface of Nuodeng ham included *Scopulariopsis*, *Epicoccum*, *Pithoascus*, *Phallu, Valsa*, *Hypochnicium*, *Pithoascus Aspergillus, Cercospora, Isaria, Penicillium*, and so on. No *P. lanosum* was detected in any of these famous hams, but *P. lanosum* was the dominant fungus in Mianning ham. At present, there are few reports on *P. lanosum*, and its effect on quality characteristics and flavor formation in ham needs to be further studied. In this study, only five kinds of microorganisms were isolated. This kind of result may be related to sample freezing treatment.

**TABLE 5:** Identification results of five strains isolated from Mianning ham.

Name	Identification results
MN1	*Penicillium lanosum*
MN2	*Penicillium nalgiovense*
MN3	*Debaryomyces hansenii*
MN4	*Staphylococcus equorum*
MN5	*Erwinia tasmaniensis*

### OTU Diversity and Taxonomic Annotation

The alpha diversity index of fungi in Mianning ham is shown in Table 6. After raw reads were obtained by sequencing, low-quality reads were filtered. Then, they were assembled and refiltered to obtain effective data for OTU clustering (Nicholas et al., 2013). A total of 95,733 high-quality ITS gene sequences were recovered from the surface, with a total length of 33,296,274 bp. The sequences were clustered into 41 OTUs with 97% consistency. A total of 97,820 high-quality ITS gene sequences were recovered internally, with a total length of 34,140,823 bp. The sequences were clustered into 43 OTUs with 97% consistency. There was a small difference in the number of OTUs from the surface and internal samples, indicating a small difference in the diversity of fungi on the surface and internal areas. Both the surface and internal fungal coverage rates were 1.00, indicating that the sequencing depth was sufficient to reflect the fungal community contained in the samples. The fungal abundance on the surface of the ham was higher than that on the internal, and the fungal diversity on the internal was higher than that on the surface.

**TABLE 6:** Alpha diversity indexes of fungi in Mianning hams.

Samples	Reads	Observed OTUs	Shannon	Simpson	Chao1	Ace	Good’s coverage
BM	95,733	41	0.31	0.07	45.00	51.20	1.00
NB	97,820	43	0.70	0.16	45.14	46.32	1.00

**FIGURE 1:**
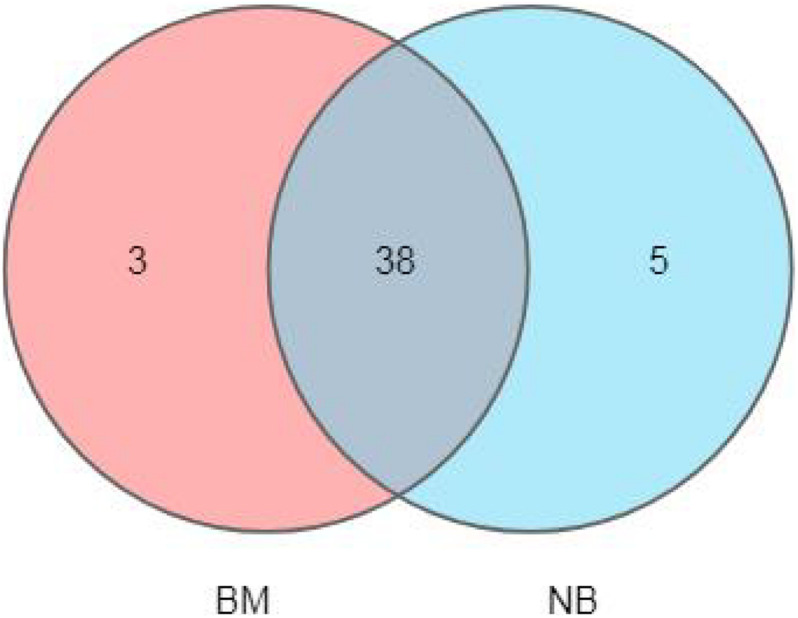
Venn diagram of fungal diversity of Mianning hams.

A Venn diagram (Figure 1) was used to analyze and compare the common and unique OTUs among and within groups to preliminarily understand the OTU characteristics among groups. Forty-one OTUs were detected on the surface, and 43 OTUs were detected internally. There were 38 common OTUs, accounting for the vast majority of the OTUs. This result indicates that the difference in the fungal community composition on the surface and internal areas is small, but the internal microbial diversity is higher than that on the surface.

### Microbial Community Composition

**FIGURE 2:**
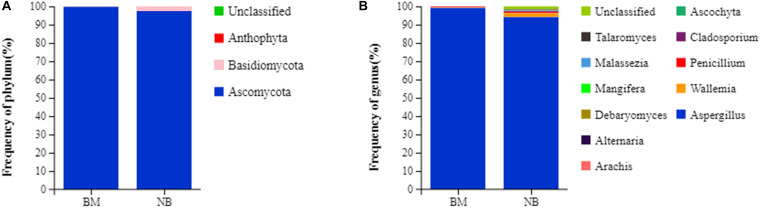
Relative abundance of fungal community proportions at the phylum **(A)** and genus **(B)** levels in Mianning ham.

Dominant species largely determine the ecological and functional structures of the microbial community. Understanding the species composition of the community at all levels can allow for effective interpretation of the formation, changes and ecological impacts of the community structure. In this experiment, the fungal community composition on the surface and internal eukaryotes of Mianning ham at the phylum and genus levels was statistically analyzed. The fungal abundance was visualized in the form of a stack diagram (Figure 2). Three phyla were detected in the surface and internal fungal communities, of which Ascomycota was the dominant phylum, accounting for 99.72 (surface) and 97.49% (internal) of the total fungal population, respectively, followed by Basidiomycota. A total of 11 genera were detected at the genus level. *Penicillium* and *Wallemia* were found to be the dominant fungal genera, accounting for 99.19 (surface) and 99.20% (internal) of the total fungal population, respectively. The dominant phylum of Panxian ham is Ascomycota, and the dominant genera are *Aspergillus* and *Penicillium*, similar to those of Mianning hams (Mu et al., 2019).

## Conclusion

Mianning ham is a specialty meat product of Sichuan province. No detailed reports have been published on Mianning ham. This study revealed the physical and chemical characteristics, microbial contents, dominant bacteria, flavor substances, and fungal community composition of Mianning ham, providing a theoretical reference for further research on these hams.

The physical and chemical indexes of Mianning ham were within the range of food safety standards, and the pH value was approximately 5.76, which promotes the presentation of flavor. A low moisture content and a_W_ are important for the preservation of Mianning ham. Mianning ham is rich in flavor compounds. A total of 60 flavor compounds were detected by SPME-GC-MS technology, 49 of which were detected on the surface and 36 in the internal. There were more kinds of flavor compounds on the surface than internally, among which aldehydes and alcohols were the most abundant. Five kinds of bacteria were isolated from Mianning ham by the traditional culture method. The identification results showed that MN1 was *P. lanosum*, MN2 was *P. nalgiovense*, MN3 was *D. hansenii*, MN4 was *S. equorum*, and MN5 was *E. tasmaniensis.* By Illumina high-throughput sequencing, the fungal diversity index showed that the internal fungal diversity was higher than that of the surface of the Mianning ham. Three fungal phyla were detected, of which Ascomycota was the dominant phylum, followed by Basidiomycota. Eleven fungal genera were detected, of which *Aspergillus* was the dominant genus, followed by *Penicillium* and *Wallemia.*

## Data Availability Statement

The datasets presented in this study can be found in online repositories. The names of the repository/repositories and accession number(s) can be found below: NCBI SRA (https://www.ncbi.nlm.nih.gov/sra/PRJNA679145).

## Author Contributions

ZW: experiment and writing—review and editing. LJ, TB, BH, and JZ: formal analysis. RZ: validation. LC: writing—original draft preparation and funding acquisition. WW: supervision. All authors contributed to the article and approved the submitted version.

## Conflict of Interest

The authors declare that the research was conducted in the absence of any commercial or financial relationships that could be construed as a potential conflict of interest.
